# Insanity as a Defence in the Criminal Courts—II

**Published:** 1908-10-24

**Authors:** 


### Medico-Legal Points.

### INSANITY AS A DEFENCE IN THE CRIMINAL COURTS—II.

Judging from the few cases that have been reported, the course of practice in the English criminal courts has been in strict conformity with the principles laid down by Lord Hale. For instance, in the trial of Arnold in 1723 for shooting at Lord Onslow, Mr. Justice Tracy observed, "That it is not every kind of frantic humour, or something unaccountable in a man's actions, that points him out to be such a madman, as is exempted from punishment; it must be a man that is totally deprived of his understanding and memory, and doth not know what he is doing, no more than an infant, than a brute, or a wild beast; such a one is never the object of punishment." This is but the echo of Lord Hale's doctrine, and the circumstances of the case show how faithfully the principles were applied.

Arnold seems to have been of weak understanding from his birth, and to have led an idle, irregular, and disordered life, sometimes unequivocally mad, and at all times considered exceedingly strange and different from other people; one witness describing him as a strange sullen boy at school, such as he had never seen before. It was testified by his family and his neighbours that for several years previous, they had considered and treated him as mad occasionally, if not always, although so little disposed to mischief that he was suffered to be at large. Contrary to the wishes of his friends, he persisted in living alone in a house destitute of the ordinary conveniences; was in the habit of lying about in barns and under hay-ricks; would curse and swear to himself for hours together; laugh and throw things about the house without any cause whatever, and was much disturbed in his sleep by fancied noises. Among other unfounded notions, he believed that Lord Onslow, who lived in his neighbourhood, was the cause of all the tumults, disturbances, and wicked devices, that happened in the country, and his thoughts were greatly occupied with this person. He was in the habit of declaring that Lord Onslow sent his devils and imps into his room at night to disturb his rest, and that he constantly plagued and bewitched him by getting into his belly or bosom, so that he could neither eat, drink, nor sleep for him. He declared in prison it was better to die than live so miserably and manifested no compunction for what he had done. Under the influence of these delusions he shot at and wounded Lord Onslow.

The proof of insanity was strong enough, but not that degree of it which the jury considered sufficient to save him from the gallows, and he was accordingly sentenced to be hanged. Lord Onslow himself, however, thought differently; and by means of his intercession, the sentence was not executed, and Arnold was continued in prison for life. It is clear that the court recognised that class of madmen only, as exempted from the penal consequences of crimes, whose reason is completely dethroned from its empire, and who are reduced to the condition of an infant, a brute, or a wild beast. It appears then, that the law at that time did not consider an insane person irresponsible for crime, in whom there remained the slightest vestige of rationality; though it did then, and has ever since, deprived him of the management of himself and his affairs, and vitiates his civil acts, even when they have no relation to the delusions that spring from his madness.

On the trial of Hadfield for shooting at the King in Drury Lane Theatre in 1800, there occurred for the first time in an English criminal court, anything like a thorough and enlightened discussion of insanity as connected with crime; and the result was, that a fatal blow was given to the doctrines of Lord Hale by Mr. Erskine, who brought all the energies of his great mind to bear upon the elucidation of this subject. In accordance with these doctrines, the attorney-general had told the jury that to protect a person from criminal responsiblity there must be a total deprivation of memory and understanding. To this Mr. Erskine very justly replied, that if these expressions were meant to be taken in the literal sense of the words—which, however, he did not admit—" then no such madness ever existed in the world." This condition of mind is observed only in idiocy and fatuity, and its unhappy subjects are never made accountable to the laws. In proper madness, on the contrary, so far was there from being a total deprivation of memory and understanding, that "in all the cases that have filled Westminster Hall," said he, "with the most complicated considerations, the lunatics and other insane persons who have been the subjects of them, have not only had memory *in my sense of the expression*, they have not only had the most perfect knowledge and recollection of all the relations they stood in towards others, and of the acts and circumstances of their lives, but have, in general, been remarkable for subtlety and acuteness. Defects in their reasonings have seldom been traceable—the disease consisting in the delusive sources of thought; all their deductions, within the scope of their malady, being founded on the *immovable* assumption of matters as *realities*, either without any foundation whatever, or so distorted and disfigured by fancy, as to be nearly the same thing as their creation." Instead, therefore, of making that kind of insanity which would exempt from punishment to consist in the absence of any of the intellectual faculties, he lays down *delusion* as its true character, of which the criminal act in question must be its immediate unqualified offspring.

Here was a great step made in this branch of medical jurisprudence, and it might have been expected, that the victory thus gained over professional prejudices and time-honoured errors would be felt in all subsequent decisions. But, though the day has gone by when such insanity only as is attended by total deprivation of memory and understanding can be admitted in excuse for crime, the test offered by Erskine was altogether too simple and too philosophical to be readily adopted by minds that delighted in subtleties and technicalities.

The case of Bellingham is the most notorious in the medico-legal annals of England. Bellingham was a delusional lunatic of much the same kind as Hadfield, except that his delusions were of the persecutory type—by far the most dangerous of all. He believed that the Government owed him a large sum of money—about £100,000—and he endeavoured to recover this by appealing to Cabinet Ministers; and he even tried to bring the matter before Parliament. He had not the shadow of a rational claim upon the Government; the whole thing was an insane delusion, and had been recognised as such by his friends for some years. He had tried to enlist the interest of Mr. Spencer Perceval, First Lord of the Treasury; and, failing in this, he had shot and killed Mr. Perceval in the lobby of the House of Commons. The doctrine of delusion, so skilfully elaborated by Erskine in Hadfield's case, was entirely discarded in the case of Bellingham. Sir James Mansfield, in charging the jury, said: "If a man were deprived of all power of reasoning, so as not to be able to distinguish whether it was right or wrong to commit the most wicked transaction, he could not certainly do an act against the law. Such a man, so destitute of all power of judgment, could have no intention at all." By this ingenious reasoning there can be no such thing as criminal insanity, the only responsible man is he who has so completely lost his power of reasoning that he is not able to entertain an intention to do anything. In other words, an insane man becomes exempt from punishment only when he becomes so insane as not to be able to commit an intentional act!

Again, the Chief Justice deliberately denied that an insane delusion is an excuse for crime. Thus: there is a species of insanity in which the patient fancies the existence of injury, and seeks an opportunity of gratifying revenge by some hostile act. If such a person is capable, in other respects, of distinguishing right from wrong, there is no excuse for any act of atrocity which he may commit under this description of derangement.

This was a complete reversal of the doctrine of insane delusion, according to which Hadfield had been acquitted twelve years before. It substituted for that test the older test of knowledge of right and wrong. Now Bellingham had himself resented in open court the imputation of insanity. He wished it known that he relied entirely upon the justice of his cause; that his killing of Mr. Perceval was right, and was justified by the fact that the Government had rejected his claim, and thus justified him in taking the law into his own hands; and this was a refutation of the charge of *malice prepense*. This characteristic insane logic showed a complete lack of the knowledge of right and wrong, in reference to the act itself; but, according to the logic of Sir James Mansfield, the right and wrong rule would not apply so long as the accused had the capacity to distinguish right and wrong in other respects.

From this standpoint Bellingham's case marks an era. It was one of the very first cases in which the right and wrong rule was announced as law, and then was not applied properly by the court. Many delusional lunatics who commit crimes in response to their delusions have inability to distinguish right and wrong in reference to that act. Their moral sense is blunted to that extent, at least. In Bellingham's case it was conspicuously so; and, like Guiteau, he attempted to argue it himself in open court. This leads to the obvious conclusion that if courts will insist upon this right-and-wrong rule, they should apply it properly and stand by it. The only question at issue is the crime charged; and the test of right and wrong should be applied to that act, and limited to it. The moral sense of no delusional lunatic is impaired equally on all subjects, any more than his intellect is deranged equally on all topics. He is to be tried on the question of his delusion, and how that impairs both his reason and his conscience. Any other method is irrelevant to the issue. Bellingham's case derives a large part of its ill repute, and deservedly, from the obtuseness of Sir James Mansfield on this very point. In that case it was also announced that the accused, in order to be exempt, must not be able to know "that murder was a crime against the laws of God and Nature." But there is no delusional lunatic who is ignorant of such an elementary principle of ethics. It would be as fair to insist that he must not know that two and two are four. Such tests do not touch the real question of his responsibility under the domination of an insane delusion.

